# Metabolic biochemical models of N_2_ fixation for sulfide oxidizers, methanogens, and methanotrophs

**DOI:** 10.1128/msystems.00748-25

**Published:** 2025-09-08

**Authors:** Meng Gao, Megan E. Berberich, Reid Brown, David M. Costello, James B. Cotner, Julian Damashek, Leila Richards Kittu, Ada Pastor, Robinson W. Fulweiler, J. Thad Scott, Amy M. Marcarelli, Keisuke Inomura

**Affiliations:** 1 Graduate School of Oceanography, University of Rhode Island 54083, Narragansett, Rhode Island, USA; 2 Department of Biological Sciences, Michigan Technological University 3968, Houghton, Michigan, USA; 3 Department of Plant and Microbial Biology, University of Minnesota-Twin Cities 172728 , Saint Paul, Minnesota, USA; 4 Department of Biological Sciences, Kent State University 170310, Kent, Ohio, USA; 5 Department of Ecology, Evolution and Behavior, University of Minnesota-Twin Cities 172734, Saint Paul, Minnesota, USA; 6 University of Bergen 1658, Bergen, Norway; 7 Department of Biology, Hamilton College 2576, Clinton, New York, USA; 8 GEOMAR Helmholtz Centre for Ocean Research 28402, Kiel, Germany; 9 Group of Continental Aquatic Ecology Research (GRECO), Institute of Aquatic Ecology, Universitat de Girona 16738, Girona, Spain; 10 Departments of Earth & Environment and Biology, Boston University 1846, Boston, Massachusetts, USA; 11 Department of Biology, Baylor University 14643, Waco, Texas, USA; Florida Atlantic University 1782, Boca Raton, Florida, USA

**Keywords:** N_2_ fixation, biochemical model, sulfide oxidizers, methanogens, methanotrophs, sediment, electron allocation, energy, CFM-CNF

## Abstract

Dinitrogen (N_2_) fixation provides bioavailable nitrogen to the biosphere. However, in some habitats (e.g., sediments), the metabolic pathways of organisms carrying out N_2_ fixation are unclear. We present metabolic models representing various chemotrophic N_2_ fixers, which simulate potential pathways of electron transport and energy flow, resulting in predictions of whole-cell stoichiometries. By balancing mass, electrons, and energy for metabolic half-reactions, we quantify the electron usage for nine N_2_ fixers. Our results demonstrate that all modeled organisms fix sufficient N_2_ for growth. Aerobic organisms allocate more electrons to N_2_ fixation and growth, yielding more biomass and fixing more N_2_, while methanogens using acetate and organisms using sulfate allocate fewer electrons. This work can be applied to investigate the depth distribution of N_2_ fixers based on nutrient availability, complementing field measurements of biogeochemical processes and microbial communities.

**IMPORTANCE:**

N_2_ fixation is an important process in the global N cycle. Researchers have developed models for heterotrophic and photoautotrophic N_2_ fixers, but there is a lack of modeling studies on chemoautotrophic N_2_ fixers. Here, we built nine biochemical models for different chemoautotrophic N_2_ fixers by combining different types of half-chemical reactions. We include three sulfide oxidizers using different electron acceptors (O_2_, NO_3_^−^, and Fe^3+^), contributing to the sulfur, nitrogen, and iron cycles in the sediment. We have two methanogens using different substrates (H_2_ and acetate) and four methanotrophs using different electron acceptors (O_2_, NO_3_^−^, Fe^3+^, and SO_4_^2−^). By modeling these methane producers and users in the sediment and their N_2_-fixing metabolic pathways, our work can provide insight for future carbon cycle studies. This study outlines various metabolic pathways that can facilitate N_2_ fixation, with implications for where in the environment they might occur.

## INTRODUCTION

Biological dinitrogen (N_2_) fixation, one of the key pathways in the global nitrogen (N) cycle, provides bioavailable N to the global biosphere (1–5). N_2_ fixation is widespread across the Earth (6) and is found in various environments, including oligotrophic oceans (7), deep seas (8), nutrient-rich coastal waters (9), and the cold Arctic (5, 10, 11). However, in some ecosystems with high organismal and habitat diversity (e.g., wetlands, seagrass meadows, estuaries), estimating N_2_ fixation rates and their drivers remains challenging (12, 13). In order to explore N_2_ fixation mechanisms, it is important to consider the N_2_ fixation reaction itself (equation 1), which shows that N_2_ fixation requires electrons and a large amount of energy (16 ATP per N_2_). As a result, sources of electrons and energy are important while considering this process. Redox and electron acceptor availability tend to shape the depth distribution of N_2_ fixers in aquatic sediments, determining where N_2_ fixation occurs. Investigating these patterns is essential for understanding how N_2_ fixation contributes to global nitrogen and carbon cycling.


(1) $$N_{2}+8e^{-}+10H^{+}+16ATP+16H_{2}O\to 2NH_{4}^{+}+H_{2}+16ADP+16P_{i}$$

Modeling studies of diazotrophic metabolism have primarily focused on N_2_ fixation by photoautotrophic cyanobacteria (14–16) and, to a lesser extent, heterotrophic bacteria (12, 17, 18). However, other “chemotrophic” energy-generating pathways can be coupled to N_2_ fixation, including methane oxidation, methanogenesis, and sulfur oxidation. Little is known about N_2_ fixation by chemoautotrophs, despite their crucial role in Earth’s biogeochemical history and continued prevalence in marine and freshwater systems, nor have models of these organisms been developed. Quantitative models can help predict potentially viable chemoautotrophic metabolic pathways to provide electrons and energy for N_2_ fixation (17, 19–22). Here, we constructed nine biochemical models for nine chemoautotrophs with different resource utilizations (Table 1). We named them cell flux models of chemotrophic nitrogen fixers (CFM-CNF).

Electrons for N_2_ fixation can be provided by some carbon (C) oxidation reactions (4, 23–25). For example, evidence shows that in methanotrophs, which are organisms that metabolize methane as their chemical energy source, methane oxidation can be coupled to N_2_ fixation (26), with electrons transferred both to N_2_ and to electron acceptors for energy production. For example, O_2_ is a favorable electron acceptor in oxic environments, while in anoxic environments, NO_3_^−^ (27), Fe^3+^ (28), and the less favorable acceptor SO_4_^2−^ (29) are all reported as electron acceptors in methanotrophs (30, 31). These various electron acceptors distinguish different types of methanotrophs: O_2_ reducers, NO_3_^−^ reducers, Fe reducers, and SO_4_^2−^ reducers. We included these four different types of methanotrophs in this modeling study to represent the variation of these electron acceptors in different habitats.

In the global carbon cycle, methanogens, which are organisms that can produce methane, also play an important role. The methane production processes are electron-accepting reactions that can reduce CO_2_ to methane and release energy. Hydrogen (H_2_) and acetate are important substrates for methanogens (32, 33). Some methanogens have also been reported as N_2_ fixers, which broadens the organisms capable of N_2_ fixation into the Archaea (34). However, more studies are needed to explore the coupling mechanism between methanogenesis and N_2_ fixation as part of a suite of possible metabolic pathways that could support N_2_ fixation. In this study, we include methanogen (acetate and H_2_ oxidizers) models to investigate the mechanism in detail.

Previous studies have also reported that sulfur oxidation, an electron-donating reaction, can be coupled with N_2_ fixation in sediments (35, 36) as an electron source. Sulfide-oxidizing microorganisms are commonly reported in sulfidic water columns (37), marine and lake sediments (38, 39), microbial mats (40), and hydrothermal vents (41). These sulfide oxidizers can use O_2_ (39), NO_3_^−^ (42, 43), and Fe^3+^ reduction (44) to provide energy, and all of these reactions have been reported to have a coupling effect with N_2_ fixation (7, 45, 46).

**Fig 1:**
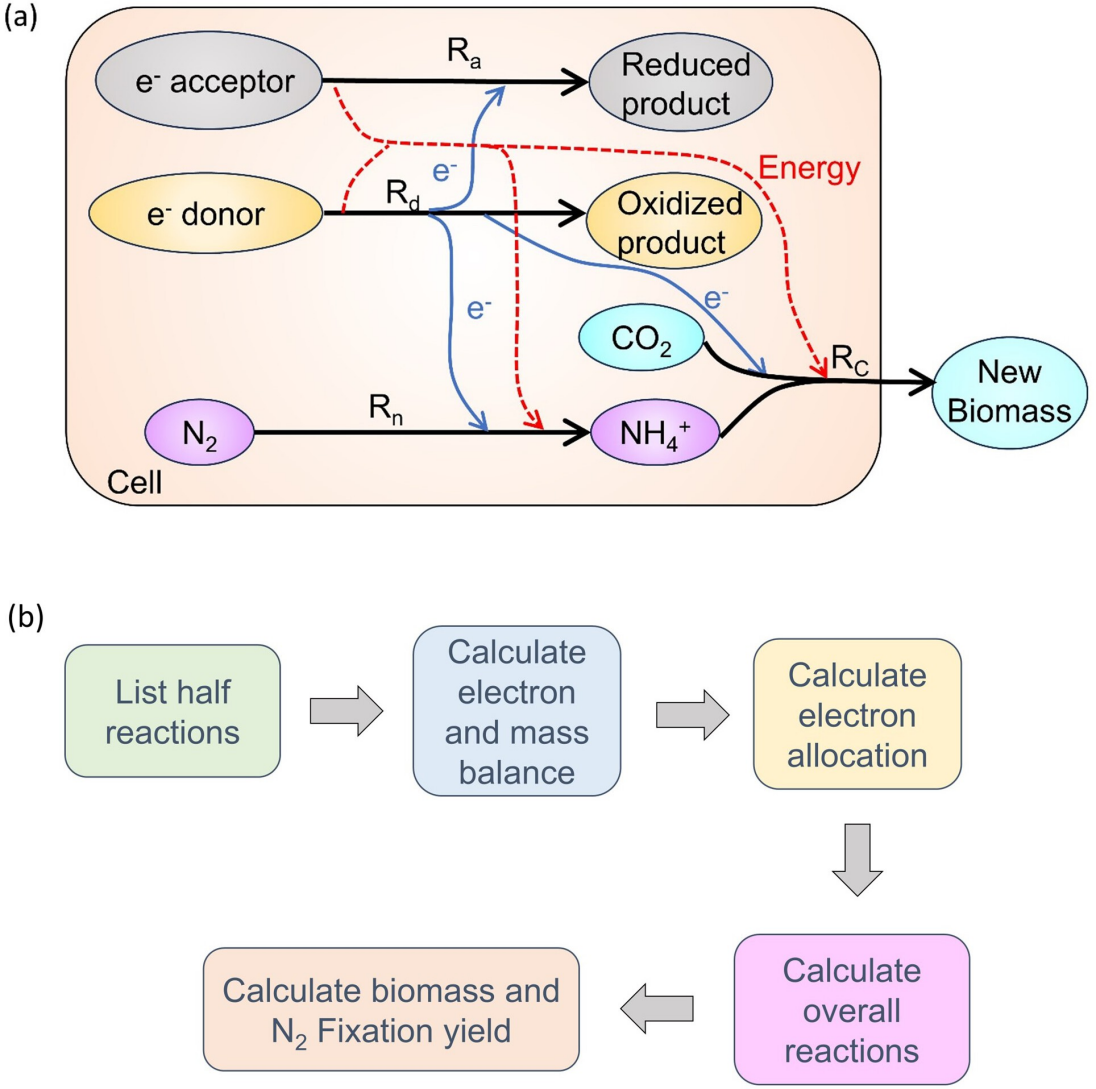
Model and simulation. (**a**) CFM-CNF schematic. Black arrows mean four main reaction types: Ra (electron acceptance), Rd (electron donation), Rn (N_2_ fixation), and Rc (biosynthesis via C fixation). Blue arrows mean electron flow from Rd to others. Red dash arrows mean energy flow. (**b**) Workflow to do the simulation. For each of the models, we listed the potential chemical reactions included, balanced the electrons and mass, and calculated the electron allocation. We added all the half reactions and calculated the overall reactions. Based on overall reactions, we calculate the biomass and N_2_ fixation yield and do the comparison.

For all of these organisms above (including methanotrophs [O_2_ reducers, NO_3_^−^ reducers, Fe^3+^ reducers, and SO_4_^2−^ reducers], methanogens [H_2_ oxidizers, and acetate oxidizers], and sulfide oxidizers [O_2_ reducers, NO_3_^−^ reducers, Fe^3+^ reducers]; Table 1), more studies are needed to investigate the potential chemical pathways or use energetic data to understand how N_2_ fixation affects their growth or ecology. Each of the nine biochemical models built in this study includes four different types of reactions (Fig. 1a): an electron donation reaction (Rd) to donate the electrons to the reductive half-reactions including synthesis and energy providers; a N_2_ fixation (Rn) half-reaction and a biosynthesis (Rc) half-reaction to fix N_2_ and grow, and an electron acceptance reaction (Ra) to provide enough energy for the N_2_ fixation and biosynthesis. The constructed models integrate both the sources and usage of electrons and energy and consider N_2_ fixation and growth, which are two essential processes in the cells of N_2_ fixers. This model framework was adapted from Rittmann and McCarty (47) and follows the fundamental laws of mass, electron, and energy conservation. Based on these energy relationships, we calculated electron allocation, biomass yield, and N_2_ fixation yield (Fig. 1b) and compared these results among the nine N_2_ fixers. Our models address the following questions. (i) What key biochemical reactions are coupled with N_2_ fixation in different chemoautotrophs? (ii) How are electrons allocated to different biochemical reactions in these organisms? (iii) How do electron acceptor efficiencies influence N_2_ fixation and growth?

**TABLE 1:** Models and the applied half reactions^*a*^

Model	Rd (donation)	Ra (acceptance)
Sulfide oxidizer (O_2_)	2	6
Sulfide oxidizer (NO_3_^−^)	2	7
Sulfide oxidizer (Fe^3+^)	2	8
Methanogen (acetate)	3	9
Methanogen (H_2_)	4	9
Methanotroph (O_2_)	5	6
Methanotroph (NO_3_^−^)	5	7
Methanotroph (Fe^3+^)	5	8
Methanotroph (SO_4_^2−^)	5	10

^*a*^ Note: equations 2 to 10 are half reactions listed in Table 2 (Rd) and (Table 3) (Ra). For all of the models, Rn (N_2_ fixation) is equation 1 listed in the introduction, and Rc (biosynthesis) is equation 11.

**TABLE 2:** A list of half reactions for electron donors (Rd)

Chemical equation	Equation number
$\frac{1}{8}\;H_{2}S+\frac{1}{2}\;H_{2}O=\frac{1}{8}SO_{4}^{2-}+\;\frac{5}{4}H^{+}+e^{-}$	2
$\frac{1}{8}\;CH_{3}COO^{-}+\frac{3}{8}\;H_{2}O=\frac{1}{8}CO_{2}+\frac{1}{8}HCO_{3}^{-}+e^{-}+H^{+}$	3
$\frac{1}{2}\;H_{2}=H^{+}+e^{-}$	4
$\frac{1}{4}\;H_{2}O+\frac{1}{8}\;CH_{4}=\frac{1}{8}\;CO_{2}+\;H^{+}+e^{-}$	5

**TABLE 3:** A list of half reactions for electron acceptors (Ra)

Chemical equation	Equation number
$\frac{1}{4}\;O_{2}+\;H^{+}+e^{-}=\;\frac{1}{2}\;H_{2}O$	6
$\frac{1}{8}NO_{3}^{-}+\frac{5}{4}H^{+}+e^{-}=\frac{1}{8}NH_{4}^{+}+\frac{3}{8}\;H_{2}O$	7
$Fe^{3+}+e^{-}=\;Fe^{2+}$	8
$\frac{1}{8}\;CO_{2}+\;H^{+}+e^{-}=\frac{1}{4}\;H_{2}O+\frac{1}{8}\;CH_{4}$	9
$\frac{1}{8}SO_{4}^{2-}+\;\frac{5}{4}H^{+}+e^{-}=$ $\frac{1}{8}\;H_{2}S+\frac{1}{2}\;H_{2}O$	10

## RESULTS AND DISCUSSION

Our study includes nine different models: sulfide oxidizers (O_2_ reducers, NO_3_^−^ reducers, Fe^3+^ reducers), methanogens (H_2_ oxidizers and acetate oxidizers), and methanotrophs (O_2_ reducers, NO_3_^−^ reducers, Fe^3+^ reducers, and SO_4_^2−^ reducers), which have all been listed in Table 1. The first step of our simulation was to determine the appropriate half-reactions (Ra, Rd, Rn, Rc) for each model.

### Overview of the half-chemical reactions

Here, we describe how we built the CFM-CNF by using four different half-chemical reactions (Table 1; Fig. S1). For sulfide oxidizers, they oxidize the sulfide to provide electrons and can use O_2_, NO_3_^−^, and Fe^3+^ as electron acceptors. So, we considered sulfide oxidation (equation 2) as Rd and included O_2_ (equation 6), NO_3_^−^ (equation 7), and Fe^3+^ reduction (equation 8) (39, 42, 43) as Ra for different types of sulfide oxidizers (Table 1). For methanogens, their key characteristic is to produce methane, so we consider the methane-producing half-reaction (equation 9) as the electron acceptance (Ra). There are different types of methanogens using different substrates to provide energy and electrons, including acetate (equation 3) and H_2_ reduction (equation 4) (48), so we use these two as the two different Rd for two methanogens. Methanotrophs oxidize methane to provide electrons and energy, so we used methane oxidation (equation 5) as the Rd for the methanotroph models. To provide enough energy, they can reduce O_2_ (equation 6), NO_3_^−^ (equation 7), Fe^3+^ (equation 8), and SO_4_^2−^ (equation 10) (27–29). We used these half reactions as Ra for different methanotrophs. The half-reactions to represent Rn and Rc are listed as equations 1 and 11, which show the general N_2_ fixation and growth processes (47).


(11) $$\frac{1}{5}CO_{2}+\frac{1}{20}NH_{4}^{+}+\frac{1}{20}HCO_{3}^{-}+\;H^{+}+e^{-}=\frac{1}{20}\;C_{5}H_{7}O_{2}N+\frac{9}{20}\;H_{2}O$$

### Electron allocation

**Fig 2:**
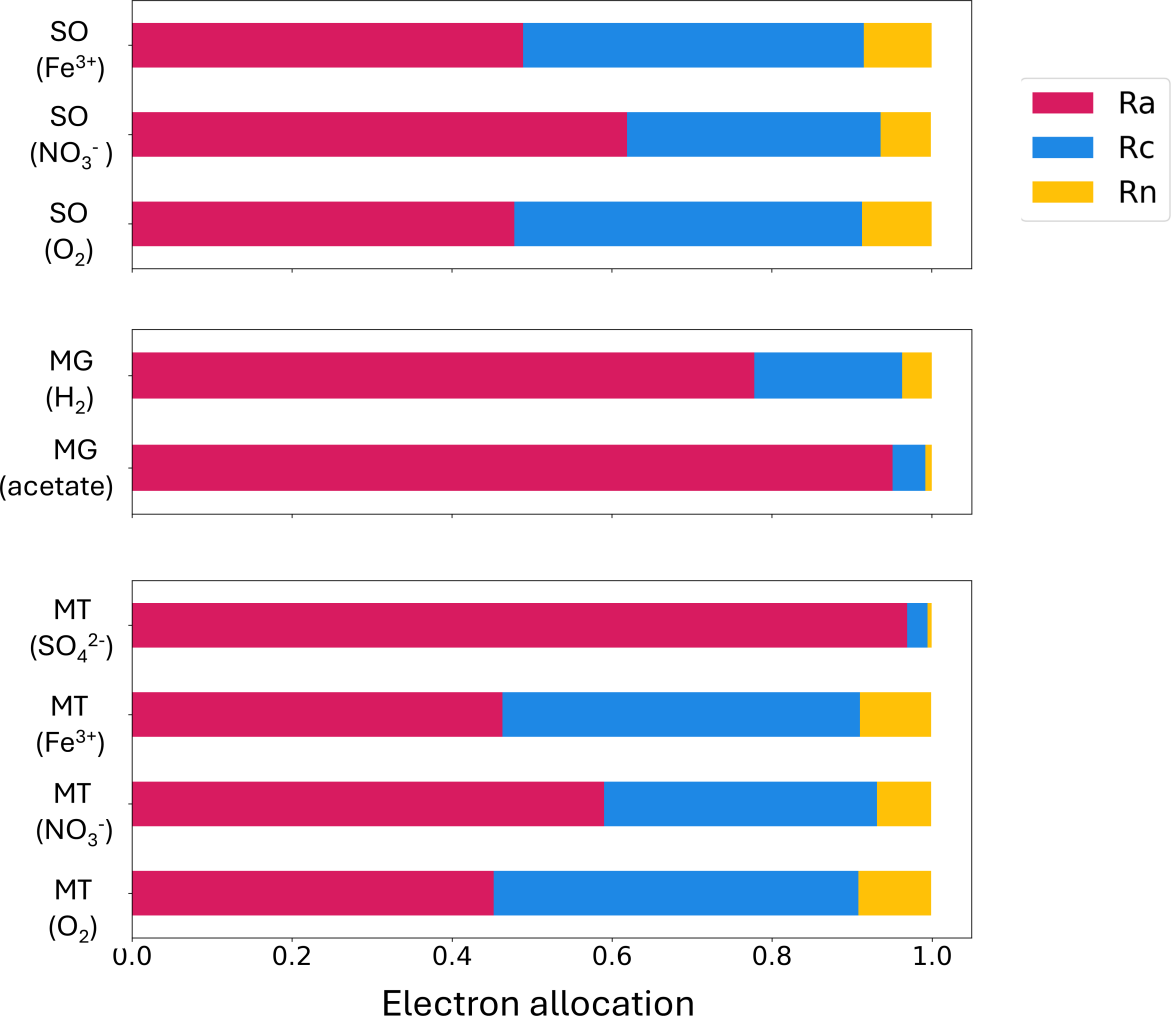
Electron allocation for different organisms. SO: sulfide oxidizers (top panel). MG: methanogens (middle panel). MT: methanotroph (bottom panel). The length of the bars represents electron usage in different reactions. Pink bars are electrons used for Ra (electron acceptance), blue bars are electrons used in Rc (biosynthesis via C fixation), and yellow bars are electrons used for Rn (N_2_ fixation). The parentheses mean different electron acceptors or donors for different models.

These half-reactions are essential to understanding how electrons are allocated to different cellular processes (e.g., N_2_ fixation) across these nine organisms. Our models simulate the electron allocations, which represent the number of electrons allocated to each half-reaction (Ra, Rc, and Rn) if there is one electron released from Rd. When we compare the allocation to Rc and Rn among different models (Fig. 2; Table S2), methanotrophs (O_2_) have the highest proportion of electrons dedicated to biosynthesis (0.456) and N_2_ fixation (0.091) (Fig. 2; Table S2). Previous evidence showed that methane oxidation under aerobic conditions can enhance N_2_ fixation (26), which is consistent with our model results. Methanotrophs (except for when SO_4_^2−^ is the electron acceptor) use more electrons in Rc and Rn than sulfide oxidizers, yet the opposite is true for Ra (Fig. 2). Evidence for methanotrophic N_2_ fixers has been reported in previous cultural and genetic studies: with the sequencing of *nifH* fragments, researchers found that both types I and II methanotrophs could fix N_2_ (49). In studies of wetlands (26) and root tissues of paddy rice (50), N_2_ fixation activity was also observed. Our results indicate the theoretical electron acceptance pathways for these methanotrophs and suggest that aerobic pathways could be those with the highest electron efficiency.

Methanogens (acetate) and methanotrophs (SO_4_^2−^) use more electrons in Ra and less in Rc and Rn. Although the electrons used in Rc and Rn are low, these values still exist, meaning these processes can still happen in methanogens (acetate) and methanotrophs (SO_4_^2−^). This is consistent with the previous studies: for methanotrophs using SO_4_^2−^, studies found that they can live with only N_2_ sources (49). For methanogens, a previous wetland study suggested that organic matter could be the electron donors for methanogens, and they can fix N_2_ (26). Our results suggest that although methane and SO_4_^2−^ used in these two organisms may not provide large amounts of energy to support biosynthesis and N_2_ fixation, their electron donation pathways, including acetate oxidation and methane oxidation, could provide enough energy for their survival and N_2_ fixation. These results indicate that in some sedimentary environments with organic C, methane, and SO_4_^2−^, N_2_ fixers can exist and may be supported by the half-reactions such as acetate oxidation, methane oxidation, methanogenesis, and SO_4_^2−^ reduction. Our result in electron allocation indicates how electrons are distributed among chemical reactions, influencing reaction rates and ultimately affecting biomass and N_2_ fixation yields.

### Overall reactions and yield: biomass and nitrogen yield per electron

**Fig 3:**
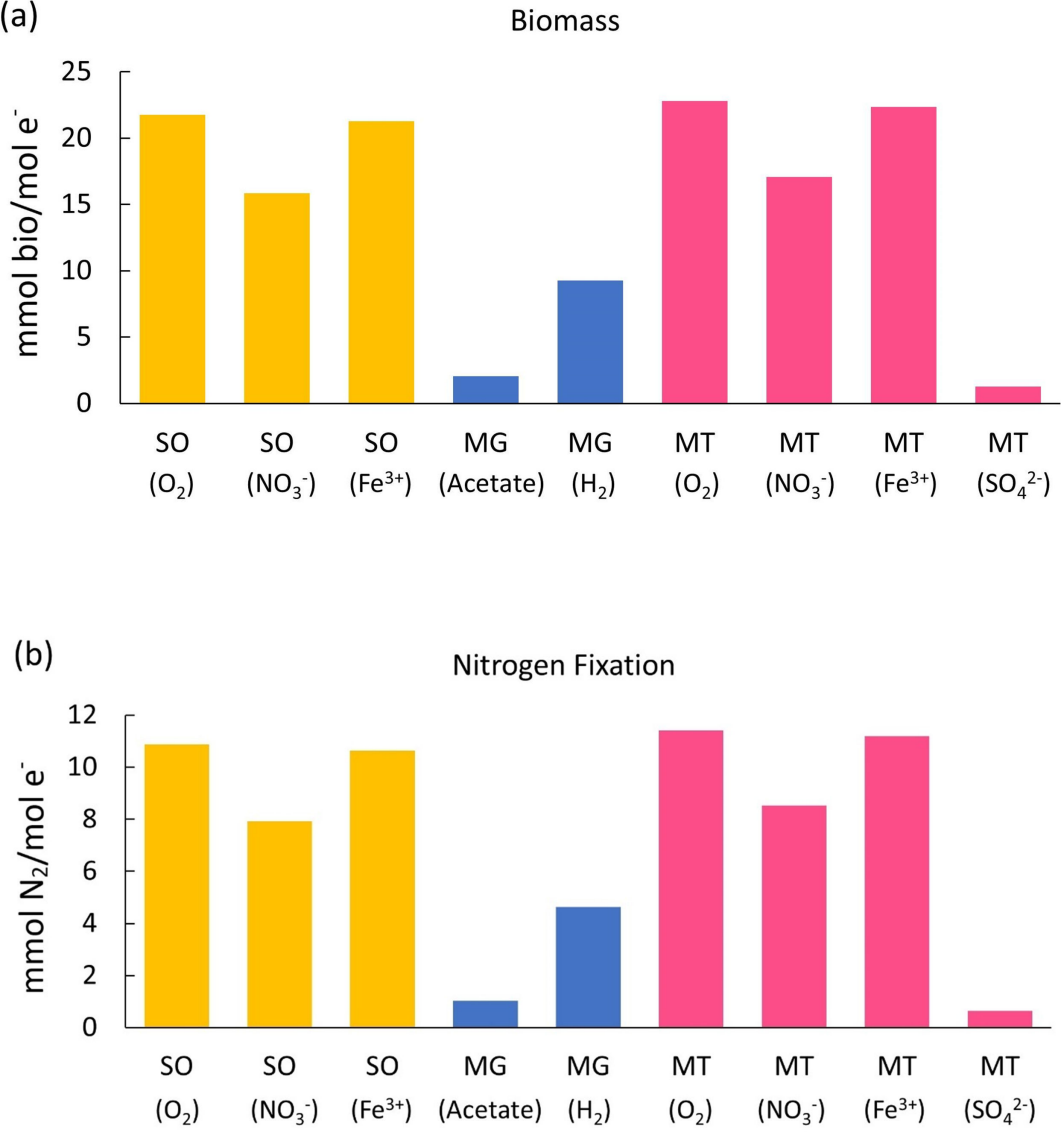
Biomass formation (**a**) and N_2_ fixation (**b**) comparison. Yellow bars represent sulfide oxidizers (SO) results, blue bars are the methanogens (MG) results, and pink bars are methanotrophs (MT) results.

Based on the half-reactions and potential electron and energy relationships listed above, we calculated the coefficient of each half-reaction and added all reactions together to obtain nine overall reaction equations (see Table S1), representing the nine different model metabolic organisms. We assume there is one transported electron for each reaction to normalize the equation. Based on these overall reactions, we can compare biomass and N_2_ fixation yields (Fig. 3; Table 4) using the coefficients of the biomass term (C_5_H_7_O_2_N) and N_2_ term (Table S1). Figure 3a and b show the amount of biomass produced and N_2_ fixed (in mmol) per mol of electron transport. In Fig. 2 and Table S1, all nine overall reactions have a positive coefficient for N_2_ as a reactant and biomass as a product, suggesting that the N_2_ fixation reaction (Rn) and biosynthesis (Rc) can be performed by all nine of these model organisms.

**TABLE 4:** Models, electron donors and acceptors, metabolic pathways, and yield

Model	e^−^ donor	e^−^ acceptor	Metabolic pathway	Biomass yield (mmol bio/mol e^−^)	N_2_ fixation yield (mmol N_2_/mol e^−^)
Sulfide oxidizer (O_2_)	H_2_S	O_2_	Oxygenic sulfide oxidation	21.76	10.88
Sulfide oxidizer (NO_3_^−^)	H_2_S	NO_3_^−^	Anaerobic sulfide oxidation	15.85	7.927
Sulfide oxidizer (Fe^3+^)	H_2_S	Fe^3+^	Anaerobic sulfide oxidation	21.28	10.64
Methanogen (acetate)	Acetate	CO_2_	Methanogenesis	2.053	1.027
Methanogen (H_2_)	H_2_	CO_2_	Methanogenesis	9.261	4.631
Methanotroph (O_2_)	CH_4_	O_2_	Aerobic methane oxidation	22.81	11.41
Methanotroph (NO_3_^−^)	CH_4_	NO_3_^−^	Anaerobic methane oxidation	17.07	8.534
Methanotroph (Fe^3+^)	CH_4_	Fe^3+^	Anaerobic methane oxidation	22.35	11.18
Methanotroph (SO_4_^2−^)	CH_4_	SO_4_^2−^	Anaerobic methane oxidation	1.276	0.638

The significance of this finding is to identify and compare the diverse metabolic pathways that may support N_2_ fixation in sedimentary systems with varied nutrient availability. Based on the availability of various electron donors and acceptors in the sediments, our research suggests the potential microbial metabolic pathways that can maintain N_2_ fixation under a range of biogeochemical conditions. These models have broader implications that N_2_-fixing communities may be highly flexible with various possible metabolic pathways, which can be adapted to changing environments. Furthermore, this model provides the basic framework for understanding nutrient cycling and microbial ecology in sedimentary environments and other environments inhabited by chemoautotrophic N_2_ fixers.

The trend of N_2_ fixation yield (Fig. 3b) in different organisms is similar to the biomass yield (Fig. 3a). This is because if more energy and electrons can be provided to biosynthesis, based on the constant fraction of biosynthesis and N_2_ fixation in synthesis reactions (equations 15 and 16), more energy and electrons can also be transported to N_2_ fixation, causing a higher N_2_ fixation yield. In the following sections, we divided the nine organisms into high-efficiency groups (yield higher biomass and N_2_ fixation, including methanotrophs [O_2_], methanotrophs [NO_3_^−^], methanotrophs [Fe^3+^], and all modeled sulfide oxidizers) and low-efficiency groups (yield lower biomass and N_2_ fixation, including methanogens and methanotrophs [SO_4_^2−^]).

### High-efficiency N_2_ fixers

When using the same electron acceptors, methanotrophs can form more biomass (methanotrophs [O_2_]: 22.81 mmol/mol e^−^, methanotrophs [NO_3_^−^]: 17.07 mmol/mol e^−^, methanotrophs [Fe^3+^]: 22.35 mmol/mol e^−^) and fix more N_2_ (methanotrophs [O_2_]: 11.41 mmol/mol e^−^, methanotrophs [NO_3_^−^]: 8.534 mmol/mol e^−^, methanotrophs [Fe^3+^]: 11.18 mmol/mol e^−^) than sulfide oxidizers (biomass: sulfide oxidizers [O_2_]: 21.76 mmol/mol e^−^, sulfide oxidizers [NO_3_^−^]: 15.85 mmol/mol e^−^, sulfide oxidizers [Fe^3+^]: 21.28 mmol/mol e^−^; N_2_ fixation: sulfide oxidizers [O_2_]: 10.88 mmol/mol e^−^, sulfide oxidizers [NO_3_^−^]: 7.927 mmol/mol e^−^, sulfide oxidizers [Fe^3+^]: 10.64 mmol/mol e^−^). For these two different electron donors, methane oxidation ($\Delta G^{0^{\prime }}=\;-23.52\;kJ/e^{-}eq$) releases more energy than sulfide oxidation ($\Delta G^{0^{\prime }}=\;-20.85\;kJ/e^{-}eq$), supporting more N_2_ fixation and growth. Previous studies have found that sulfide oxidizers and N_2_ fixers in aquatic systems and sediment (36, 45), with the electron acceptors O_2_ and NO_3_^−^ . Although we haven’t found an experimental comparison in N_2_ fixation yield between sulfide oxidizers and methanotrophs, our study calculated these theoretical values and fills the gap.

We can also compare the yields among models with different electron acceptors. In our results, O_2_ is the best electron acceptor yielding more biomass (Fig. 3a; Table 4, sulfide oxidizer [O_2_]: 21.76 mmol/mol e^−^, methanotrophs [O_2_]: 22.81 mmol/mol e^−^) and fixing more N_2_ (Fig. 3b; Table 4, sulfide oxidizer [O_2_]: 10.88 mmol/mol e^−^, methanotroph [O_2_]: 11.41 mmol/mol e^−^) than other electron acceptor models. According to the energy state of reactions, O_2_ reduction releases more energy ($\Delta G^{0^{\prime }}=\;-78.72\;kJ/e^{-}eq$) per electron transport, which provides more energy for growth and N_2_ fixation. These efficient aerobic N_2_ fixers have been found in soil (49) and fish gills (45). Our models can be used to predict the metabolic pathways, biomass, and N_2_ fixation yield of these organisms.

### Low-efficiency N_2_ fixers

In our simulation results (Table S1), N_2_ fixation can be performed by all nine of these model organisms, including some less efficient N_2_ fixers, e.g., methanotrophs (SO_4_^2−^). Figure 3 shows that SO_4_^2−^ is a less effective acceptor, which can only form 1.276 mmol bio/mol e^−^ and fix 0.638 mmol N_2_/mol e^−^. Although it is less favorable ($\Delta G^{0^{\prime }}=\;20.85\;kJ/e^{-}eq$), it can still happen mathematically, which provides a quantitative explanation for the existence of SO_4_^2−^ reduction in methanotrophs. This result is also consistent with field studies, which show that methanotrophic sulfate reducers exist (35) and can inhabit the sulfate–methane interface. In a recent study in cold seeps, researchers found that sulfate reducers could utilize anaerobic methane oxidation to support N_2_ fixation (51).

Methanogens (acetate) can form less biomass ( 2.053 mmol bio/mol e^−^) and fix less N_2_ (1.027 mmol N_2_/mol e^−^) than most other modeled metabolic organisms, except for methanotrophs (SO_4_^2−^) (Fig. 3; Table 4, 1.276 mmol bio/mol e^−^ and 0.638 mmol N_2_/mol e^−^). Methanogens (H_2_) have a higher yield in biomass (9.261 mmol bio/mol e^−^) and N_2_ fixation (4.631 mmol N_2_/mol e^−^) than methanogens (acetate), which is because H_2_ oxidation is more favorable ($\Delta G^{0^{\prime }}=-39.87\;kJ/e^{-}eq$) and can provide more energy than methane oxidation. As a result, methanogens (H_2_) are more effective than methanogens (acetate). Previous studies in soil have found the *nif* gene in these two methanogens, contributing to the N production in the system (48). In their experiment, researchers found that the addition of H_2_ and CO_2_ could increase the nitrogenase activity and methane production significantly, while the addition of acetate did not have a significant effect, which is consistent with our model that H_2_ is a better electron donor in methanogen N_2_ fixers. Researchers also found that these N_2_-fixing methanogen strains can exist in some O_2_-limited environments, including marine sediments and paddy soil (52). In the methanogen (acetate) and methanotroph (SO_4_^2−^) models, Ra and Rd release less energy, leading to higher electron allocation to Ra to provide more energy and a lower allocation to Rc and Rn, resulting in less fixed N_2_ and growth.

### Effect of pH and temperature

**Fig 4:**
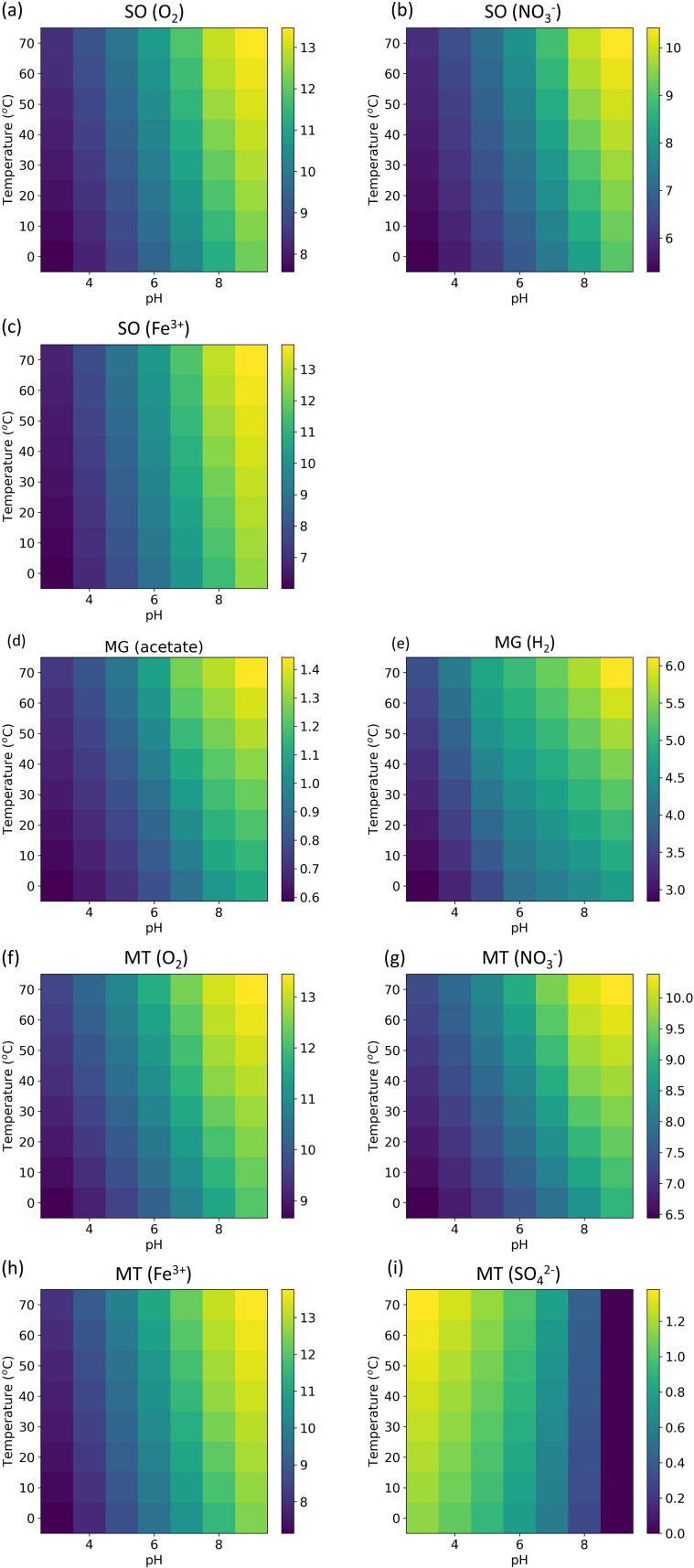
The effect of pH and temperature on N_2_ fixation yield (unit: mmol N_2_/mol e^−^) for different N_2_ fixers (sulfide oxidizers [SO] using O_2_ (**a**), NO_3_^−^ (**b**), and Fe^3+^ (**c**); methanogens [MG] using acetate (**d**) and H_2_ (**e**); Methanotrophs [MT] using O_2_ (**f**), NO_3_^−^ (**g**), Fe^3+^ (**h**), and SO_4_^2−^ (**i**)). Color means the N_2_ fixation yield (mol N_2_/mol e^−^).

Temperature and pH are both important in biochemical reactions because they directly affect reaction rates and enzyme activity. Higher temperature increases the movement and collision frequency, raising the chance of reactants overcoming the activation energy barrier. Higher temperatures can also provide more energy for N_2_ fixation and biosynthesis, facilitating the processes of these reactions. pH can affect chemical reactions directly and modulate enzyme activities. In our model, we tested the effects of temperature and pH (Fig. 4), and we found that in most of the modeled organisms, with the increase in temperature and pH, the N_2_ fixation yield increased. This trend may be explained by Le Chatelier’s principle (53): when a system’s equilibrium is disturbed, it adjusts to counteract the change and restore equilibrium. In the overall reactions for sulfide oxidizers, methanogens, and methanotrophs (Fe^3+^) (Table S1), since H^+^ is on the product side of the overall reactions, decreasing H^+^ (higher pH) drives the reaction forward to produce more H^+^, facilitating the overall reactions and fixing more N_2_ and growing more biomass. For the methanotrophs (O_2_ and NO_3_^-^), although H^+^ occurs on the reactant side of the overall reactions, H^+^ could inhibit methane-producing processes, which are the key electron donation processes to support N_2_ fixation. For methanotrophs (SO_4_^2-^), decreased pH can facilitate their N_2_ fixation because H^+^ occurs on the reactant side of their overall reaction.

### Comparison with previous studies: empirical evidence of N_2_ fixation for sulfide oxidizers, methanogens, and methanotrophs

Our predictive models based on physical, biochemical, and mathematical principles showed that N_2_ fixation could occur alongside biosynthesis in all nine organisms analyzed in this study. Our theoretical findings are supported by previous studies using empirical techniques with varying confidence levels to examine these potential reactions in different habitats. For example, research on sulfide oxidizers in fish gills identified bacteria in the genus *Candidatus* Thiodiazotropha, an aerobic symbiont with the *nif* gene for N_2_ fixation (45), which could be applied to our model for sulfide oxidizers (O_2_). Additionally, in cold seep ecosystems, researchers found that *Dechloromonas* sp. carried genes related to N_2_ fixation (*nif*DHK), sulfur compound oxidation (*fccAB* and *soxABCXYZ*), and nitrate reduction (*napAB* and *nirBD*) (54), which could be applied to the sulfide oxidizer (NO_3_^−^) model. Some N_2_ fixers, such as *Thiobacillus ferrooxidans*, a sulfide oxidizer living in low pH habitats, were found to fix N_2_ (55, 56) and, in some cases, reduce Fe^3+^ (57), which may be related to our sulfide oxidizer (Fe^3+^) models.

For methanotrophs, N_2_ fixation can also be confirmed based on N isotope (26, 50) and genetic analysis (49). Auman found that aerobic methanotrophs, *Methylococcus* spp., and sulfide-reducing methanotrophs, *Methylosinus* spp., from soils can fix N_2_ (49). Dekas et al. observed that seep N_2_ fixation is methane-dependent, and that N_2_ fixation rates peak in narrow sediments because of anaerobic methanotrophic archaea and sulfate-reducing bacteria, which form symbionts and fuel the complex ecosystems (58). Cui et al. (26) found that nitrate-reducing methanotrophs, *Methylocystis* spp., in wetlands can fix N_2_. In paddy soil under hypoxia, Yu et al. (59) found that methane oxidation coupled with iron reduction can significantly increase the biological N_2_ fixation rate, which can happen in *Methylocystis*, *Methylophilaceae*, and *Methylomicrobium*. The evidence above can, respectively, fit models of methanotroph (O_2_), methanotroph (SO_4_^2−^), methanotroph (NO_3_^−^), and methanotroph (Fe^3+^).

Methanogens (*Methanococcus maripaludis* and *Methanococcus thermolithotrophicus*, oxidizing H_2_) in lab studies without N-amended media suggested fixed N_2_ was the sole N source (60), which is consistent with our methanogen (H_2_) model. Genetic analysis also shows that methanogens can fix N_2_ (61), for example, in a wetland soil incubation study, researchers found that methanogens using H_2_ and acetate both contain N_2_ fixation genes (*nifH*) (48), which confirms our findings. All these organisms and symbionts we listed above are commonly active in many aquatic and terrestrial ecosystems, particularly in locations with oxygen gradients or highly anoxic environments, for example, sulfide oxidizers in the redoxcline of the Black Sea (62) and methanogens in marine sediments (63).

### Model construction and comparison with different model types

Our model is a coarse-grained model (4), including some important biochemical processes inside the cells. Our model integrates both the sources and utilization of electrons and energy, focusing on two fundamental cellular processes in nitrogen-fixing organisms: N_2_ fixation and growth. This modeling framework is adapted from Rittmann and McCarty (47) and follows fundamental mass, electron, and energy conservation principles. For example, when considering half-reactions, we ensure that the energy-providing reactions provide sufficient energy for the overall process to proceed spontaneously. As for electron transport, the model includes both electron donors and acceptors. Various acceptence reactions include energy generation, biomass growth, and N_2_ fixation. Additionally, the model ensures that the mass of all chemical elements involved is conserved throughout the reactions.

Our coarse-grained model lies between simple equation models and detailed metabolic models. Different modeling approaches vary in detail and scope, each with strengths and limitations suited to specific applications. Detailed metabolic models can include numerous reactions based on omics data (64). However, they typically require constraints that are often difficult to validate (65–68). On the other end, highly simplified models like Monod kinetics (69) are highly empirical yet may lack mechanistic processes (70). Coarse-grained models offer a middle-ground solution by resolving key physiological processes while remaining practical, flexible, and empirical (71).

In this study, we chose a coarse-grained model because it focuses on key cellular processes—such as electron donation and acceptance, and the budgets of mass, electrons, and energy—without relying on sets of poorly constrained parameters. We built our models upon an established method of electron allocation, which has been developed by experimental evidence (47). Although there is a possibility of increasing the detail, our level of simplification is deliberate and helps maintain consistency with available empirical data. It also improves computational efficiency and makes the model more broadly applicable across different organisms and environments. This model also provides a single solution rather than multiple possible outcomes, as often seen in more complex approaches like flux balance analysis. By aligning model complexity with data availability, we aim to keep our framework both reliable and widely usable, building on the success of similar approaches in previous studies in both Inomura groups (4, 14, 17, 19, 72) and other groups (6, 73–75).

### Future research

#### Temporal dynamics

Our model begins with steady-state conditions instead of temporal dynamics. Because we are aiming at a steady sedimentary environment, where conditions do not change significantly on the time scale of minutes and hours. The steady-state assumption has been made in multiple previously published models, including most of the detailed metabolic networks (21, 22, 76), and our study followed these examples. Regardless, future research may resolve temporal dynamics to explore the shift in chemoautotrophic N_2_ fixation in a longer time series.

#### Depth distribution

Our models help to clarify the theoretical biochemical principles behind N_2_ fixation metabolism, which can be used to understand the spatial ecological niche of various chemoautotrophic N_2_ fixers. The availability and favorability of the reduction of the electron acceptors may interact to create a depth distribution of different N_2_ fixers along oxic-anoxic gradients in sediments. For example, NO_3_^−^ reduction is more favorable than SO_4_^2−^ reduction, resulting in NO_3_^−^ reducers being found in the upper layer of the sediment (77, 78). In river estuarine sediments, SO_4_^2−^ reduction was also reported to have a potential relationship with N_2_ fixation (79). Specifically, sampling from different sediment depths combined with transcriptomics and proteomics could further validate and expand our modeling results.

### Conclusions

We constructed a whole-cell reaction stoichiometry of putative chemoautotrophic N_2_-fixing organisms. The results show a range of electron allocation based on the energetics of the half-reactions. All these putative metabolic organisms allow N_2_ fixation and biosynthesis to occur, and thus, our work uncovers the potential reactions coupled with N_2_ fixation. Based on the electron allocation results, O_2_ is a better electron acceptor because chemotrophs using O_2_ can allocate more electrons to N_2_ fixation and growth. From the biomass and N_2_ fixation yield, we found that aerobic N_2_ fixers are more efficient, while SO_4_^2−^ reducers and methanogens (acetate) are less efficient. However, even less favorable substrates, SO_4_^2−^ and CH_4_, can support N_2_ fixation. The variation between different organisms allows for the consumption of various substrates in the different catabolic reactions supporting N_2_ fixation, which may occur at different oxidative/reductive environments in aquatic systems at different depths. It is challenging to precisely measure the metabolisms of microorganisms living in sediments under variable environmental conditions. Therefore, our model will be useful in investigating the likely depth-dependent metabolic pathways for different substrate compositions. These predictions can complement field measurements by providing estimates of the contribution of chemoautotrophs to N_2_ fixation.

## MATERIALS AND METHODS

Our CFM-CNF follows an original cell flux model (CFM) structure, which simulates *Azotobacter vinelandii* (17), an extensively studied soil-dwelling N_2_ fixer. We targeted organisms inhabiting sediments, where O_2_ concentrations are typically low. So, we assume that the O_2_ concentration is low enough that we can neglect the protection from O_2_ (i.e., the respiratory protection), especially for those anaerobes using non-oxygen substrates for electron acceptors living in anoxic environments. Since we did not target the heterotrophic organisms living in highly oxic environments (e.g., those in water columns), we did not include O_2_ inhibition in this model.

This model summarizes the cell metabolism and combines four biochemical reactions: electron donation (Rd), electron acceptance for non-synthesis purposes such as energy generation (Ra), biosynthesis (Rc), and N_2_ fixation (Rn). This model can be used to calculate electron flow, biomass, and N_2_ fixation yield. Here, we explained the calculation method step by step.

### Energy reactions

Microorganisms obtain their energy for growth and maintenance from oxidation-reduction reactions, involving an electron donation half-reaction (Rd) and an electron acceptance half-reaction (Ra). For different potential N_2_ fixers in this study, we used different Rd and Ra (Table 1). We listed the Gibbs free energy change ($\Delta G^{0^{\prime }}$) for different Rd and Ra (Table S5), which describes their favorability under standard conditions. Based on their energy change, we then decide the energy-providing reactions and potential energy flow within the model.

### Biosynthesis reactions

Bacterial metabolic pathways involve two types of basic reactions, one for energy (mentioned in the last section) and the other for cellular synthesis (e.g., growth and maintenance). In this step, we need to consider the half-reactions for the synthesis part. For the growth of cells (Rc), we used equation 11. Since the purpose of this study is to model N_2_ fixers, here we also consider N_2_ fixation as a part of the maintenance of the cell (equation 1).

### Electron allocation

When microorganisms use an electron-donor substrate for synthesis, a portion of their electrons ($f_{e}$) is initially transferred to the electron acceptor to provide energy. Other portions of electrons can be transferred to growth and maintenance ($f_{s}$). The sum of $f_{e}$ and $f_{s}$ is 1 since we assumed one electron was transferred in this study.

Then, we calculated the allocation of electrons following the method in reference 47 (equations 12 to 14). Firstly, we calculated a parameter A (equation 12, derivation in the supplemental material) representing the number of electron donors that must be oxidized to supply the energy needed for cell synthesis. In equation 12, $∆G_{n}$ means Gibbs free energy change for N_2_ fixation, $∆G_{p}$ means the energy required to convert the carbon source to the common organic intermediates (activated acetate) that cells use for synthesizing their macromolecules, and $∆G_{pc}$ means the energy used to convert the organic intermediates to cellular carbon. We used 18.8 kJ/e^−^ eq as $∆G_{pc}$. ε is energy transfer efficiency. Rittmann and McCarty (47) reported that with the optimum conditions, transfer efficiencies of 55% to 70% are typical for most anaerobic and chemoautotrophic reactions, and a ε value of 0.6 is frequently employed to provide accurate results. So here we used ε equals to 0.6 for all the chemoautotrophs.

Here, an exponent n is used for the calculation of ε, according to Rittmann and McCarty (47), if $∆G_{p}$ is positive, *n* is 1; if is $∆G_{p}$ is negative, *n* is −1. $∆G_{r}$ was calculated by using Gibbs free energy change for the electron acceptance reaction (Ra) and Gibbs free energy change in the electron donation reaction (Rd).


(12) $$A=-\frac{\left(f_{n}\times \Delta G_{n}+f_{c}\times \left(\frac{\Delta G_{p}}{\varepsilon^{n}}+\frac{\Delta G_{pc}}{\varepsilon }\right)\right)}{\varepsilon \Delta G_{r}}.$$

We used the *A* value to calculate electron allocation to cell synthesis (equation 13, $f_{S}$, including Rc and Rn) and energy production (equation 14, $f_{e}$). The derivation of equation 13 and equation 14 is in the supplemental material (equations S7 to S12).


(13) $$f_{S}=\;\frac{1}{1+A}$$

(14) $$f_{e}=\;\frac{A}{1+A}$$

Here, we explain the equations we used to calculate the fraction of biosynthesis and N_2_ fixation. In equation 15, $f_{c}$ means the fraction of electrons allocated to Rc over the total electrons allocated to Rc and Rn. $Y_{bio}^{e^{-}:N}$ means the transferred electrons to biomass N ratio in Rc, and $Y_{N_{2}fix}^{e^{-}:N}$ means the transferred electrons to fixed N ratio in Rn. We calculated the ratio of Rc ($Y_{bio}^{e^{-}:N}$) to the sum of Rc and Rn ($Y_{bio}^{e^{-}:N}+Y_{N_{2}fix}^{e^{-}:N}$) to obtain this electron allocation fraction of Rc ($f_{c}$). In equation 16, the calculation is similar; we calculated this fraction of Rn ($f_{n}$) out of the sum of Rn and Rc by using the electron ratio of Rn ($Y_{N_{2}fix}^{e^{-}:N}$) to the sum of Rc and Rn ($Y_{bio}^{e^{-}:N}+Y_{N_{2}fix}^{e^{-}:N}$).


(15) $$f_{c}=\;\frac{Y_{bio}^{e^{-}:N}}{Y_{bio}^{e^{-}:N}+Y_{N_{2}fix}^{e^{-}:N}}$$

(16) $$\;\;\;\;\;\;\;\;\;\;\;f_{n}=\;\frac{Y_{N_{2}fix}^{e^{-}:N}}{Y_{bio}^{e^{-}:N}+Y_{N_{2}fix}^{e^{-}:N}}$$

### Overall reactions and yield calculation

The mass is conserved in each half-reaction. We combine these reactions to balance electrons and energy. In all the cases in this study, the combination of Rd and Ra leads to energy production, which is, in turn, used for biosynthesis (Rc) and N_2_ fixation (Rn).

Equation 17 shows the calculation of Re (the overall reactions for energy). It can be calculated from the difference between Ra and Rd.


(17) $$R_{e}=R_{a}-R_{d}$$

The synthesis reactions (Rs) (equation 18) are calculated from the difference between synthesis half reactions (Rsh) and donation (Rd).


(18) $$R_{s}=R_{sh}-R_{d}$$

Here, we considered Rn and Rc as two different sections of the synthesis half reactions (Rsh). To calculate Rsh, we multiplied Rc by a fraction of Rc ($f_{c}$) as the biosynthesis section. We multiplied Rn by a fraction of Rn ($f_{n}$) as the N_2_ fixation section.


(19) $$R_{sh}=f_{c}R_{c}+f_{n}R_{n}$$

Then we substitute $R_{sh}$ into equation 18, yielding equation 20.


(20) $$R_{s}=f_{c}R_{c}+f_{n}R_{n}-R_{d}$$

Finally, we estimated the overall reaction, which is the sum of energy reactions (Re) and synthesis reactions (Rs). Here, we calculated this by using equation 21. We multiplied Re by the electron fractions to energy ($f_{e}$) and Rs by the electron fractions to synthesis ($f_{s}$).


(21) $$R=\;f_{e}\times R_{e}+\;f_{s}\times R_{s}$$

We finally substituting equations 18 and 20 into equation 21 yields the final equation, and calculated the overall reactions (equation 22). The overall reactions for different modeling organisms are presented in Table S1. Based on the coefficients of the overall reactions, we can compare the yield for N_2_ fixation and biosynthesis. The coefficients before the N_2_ term mean the N_2_ fixed per electron, while the coefficients before the biomass term mean the biomass produced per electron.


(22) $$R=\;f_{e}\times \left(R_{a}-R_{d}\right)+\;f_{s}\times \left(f_{c}R_{c}+f_{n}R_{n}-R_{d}\right)$$

The specific values we used to do the calculations (Table S3) are all from Rittmann and McCarty (47). For different models, we have different values, which have been listed in the supplementary parameter tables (Tables S3 through S6).

## Data Availability

The data sets generated and/or analyzed during the current study are available in the "Biochemical model for sediment N_2_ fixers" repository at https://doi.org/10.5281/zenodo.12680697.

## SUPPLEMENTAL MATERIAL

Supplemental Material msystems.00748-25-s0001.pdf Tables S1 to S6; Fig. S1.

ASM does not own the copyrights to Supplemental Material that may be linked to, or accessed through, an article. The authors have granted ASM a non-exclusive, world-wide license to publish the Supplemental Material files. Please contact the corresponding author directly for reuse.
