# Supplementary material for: Metabolic biochemical models of N2 fixation for sulfide oxidizers, methanogens, and methanotrophs
Source: mSystems. 2025 Sep 8;10(10):e00748-25. doi: 10.1128/msystems.00748-25 (PMC12542792; doi:10.1128/msystems.00748-25)
Supplement: Supplemental Material — Tables S1 to S6; Fig. S1. [file msystems.00748-25-s0001.pdf]

Table S1. Resultant whole cell reactions

Models	Whole reactions
Sulfide oxidation (O_2)	$0.125\ H_2S + 0.06527\ H_2O + 0.08703\ CO_2 + 0.1195\ O_2 + 0.02176\ HCO_3^- + 0.01088\ N_2 \rightarrow 0.01088\ H_2 + 0.02176\ C_5H_7O_2N + 0.125\ SO_4^{2-} + 0.2282\ H^+$
Sulfide oxidation (NO_3^-)	$0.125\ H_2S + 0.125\ H_2O + 0.06341\ CO_2 + 0.07744\ NO_3^- + 0.01585\ HCO_3^- + 0.007927\ N_2 \rightarrow 0.01585\ C_5H_7O_2N + 0.007927\ H_2 + 0.07927\ H^+ + 0.125\ SO_4^{2-} + 0.07744\ NH_4^+$
Sulfide oxidation (Fe^{3+})	$0.125\ H_2S + 0.3085\ H_2O + 0.08512\ CO_2 + 0.4893\ Fe^{3+} + 0.02128\ HCO_3^- + 0.01064\ N_2 \rightarrow 0.02128\ C_5H_7O_2N + 0.01064\ H_2 + 0.7179\ H^+ + 0.125\ SO_4^{2-} + 0.4893\ Fe^{2+}$
Methanogen (acetate)	$0.125\ CH_3COO^- + 0.119\ H_2O + 0.00205\ CO_2 + 0.001027\ N_2 \rightarrow 0.002053\ C_5H_7O_2N + 0.123\ HCO_3^- + 0.001\ H_2 + 0.00205\ H^+ + 0.119\ CH_4$
Methanogen (H_2)	$0.495\ H_2 + 0.134\ CO_2 + 0.009261\ HCO_3^- + 0.004631\ N_2 \rightarrow 0.009261\ C_5H_7O_2N + 0.2778\ H_2O + 0.00926\ H^+ + 0.09722\ CH_4$
Methanotroph (O_2)	$0.125\ CH_4 + 0.1131\ O_2 + 0.\ 02281\ H^+ + 0.02281\ HCO_3^- + 0.01141\ N_2 \rightarrow 0.01141\ H_2 + 0.02281\ C_5H_7O_2N + 0.1816\ H_2O + 0.03375\ CO_2$
Methanotroph (NO_3^-)	$0.125\ CH_4 + 0.\ 1647\ H^+ + 0.01707\ HCO_3^- + 0.008534\ N_2 + 0.07379\ NO_3^- \rightarrow 0.008534\ H_2 + 0.01707\ C_5H_7O_2N + 0.125\ H_2O + 0.05673\ CO_2 + 0.07379\ NH_4^+$
Methanotroph (Fe^{3+})	$0.125\ CH_4 + 0.02235\ HCO_3^- + 0.01118\ N_2 + 0.4635\ Fe^{3+} + 0.04882\ H_2O \rightarrow 0.01061\ H_2 + 0.02235\ C_5H_7O_2N + 0.03559\ CO_2 + 0.4635\ Fe^{2+} + 0.\ 4412\ H^+$
Methanotroph (SO_4^{2-})	$0.125\ CH_4 + 0.001276\ HCO_3^- + 0.000638\ N_2 + 0.1212\ SO_4^{2-} + 0.2436\ H^+ \rightarrow 0.000638\ H_2 + 0.001276\ C_5H_7O_2N + 0.1198\ CO_2 + 0.1212\ H_2S + 0.2462\ H_2O$

Table S2 Electron allocation in different models (related to Figure 2, results of electron allocation calculation)

Models	Ra	Rc	Rn
Sulfide oxidation (O_2)	0.478	0.435	0.087
Sulfide oxidation (NO_3^-)	0.619	0.317	0.063
Sulfide oxidation (Fe^{3+})	0.489	0.426	0.085
Methanogen (acetate)	0.951	0.041	0.008
Methanogen (H_2)	0.778	0.185	0.037
Methanotroph (O_2)	0.452	0.456	0.091
Methanotroph (NO_3^-)	0.590	0.341	0.068
Methanotroph (Fe^{3+})	0.463	0.447	0.089
Methanotroph (SO_4^{2-})	0.969	0.0255	0.005

Table S3. Parameters and values (1) used in model

Parameters	Units	Definitions	Values
f_c	Dimensionless	The ratio of Rc to Rc plus Rn	0.833
$Y_{bio}^{e^-:N}$	Dimensionless	Electron to N ratio in biosynthesis	20
$Y_{N_2fix}^{e^-:N}$	Dimensionless	Electron to N ratio in N_2 fixation	4
f_n	Dimensionless	The ratio of Rn to Rc plus Rn	0.167
A	Dimensionless	the equivalents of electron donor must be oxidized to supply the energy need for an equivalent of cells synthesizing	Table S6
ΔG_n	kJ/e^-eq	Gibbs free energy change for N_2 fixation	Table S4
ΔG_p	kJ/e^-eq	energy required to convert the carbon source to pyruvate	Table S4 (1)
ΔG_{pc}	kJ/e^-eq	energy used to convert pyruvate	18.8145 (1)

		carbon to cellular carbon	
ΔG_r	$kJ/e^{-}eq$	Energy change in electron donor reaction	Table S4
ε	Dimensionless	energy-transfer efficiency	0.6 (1)
f_S	Dimensionless	the fraction of cell synthesis	Table S6
f_e	Dimensionless	The fraction of energy production	Table S6

Table S3 summarizes the values used in the model. $Y_{bio}^{e^{-}:N}$ and $Y_{N_2fix}^{e^{-}:N}$ represent the electron- to-N ratio in reaction [1] and [11] in the main text, indicating how many N atoms react when one electron is transported. f_c and f_n are calculated from equation [15] and [16] from the main text, representing the ratio of biosynthesis or N_2 fixation to the total synthesis

$$N_2 + 8e^{-} + 10H^{+} + 16ATP + 16H_2O \rightarrow 2NH_4^{+} + H_2 + 16ADP + 16P_i \qquad \text{Main text [1]}$$

$$\frac{1}{5}CO_2 + \frac{1}{20}NH_4^{+} + \frac{1}{20}HCO_3^{-} + H^{+} + e^{-} = \frac{1}{20}C_5H_7O_2N + \frac{9}{20}H_2O \qquad \text{Main text [11]}$$

$$f_c = \frac{Y_{bio}^{N:e^{-}}}{Y_{bio}^{N:e^{-}} + Y_{N_2fix}^{N:e^{-}}} \qquad \text{Main text [15]}$$

$$f_n = \frac{Y_{N_2fix}^{N:e^{-}}}{Y_{bio}^{N:e^{-}} + Y_{N_2fix}^{N:e^{-}}} \qquad \text{Main text [16]}$$

Table S4. Energy Values (unit: $kJ/e^{-}eq$)

Model Types	ΔG_n	ΔG_p (1)	ΔG_r
SO (O_2)	87.49	10.05	-99.57
SO (NO_3^-)	87.49	10.05	-55.96
SO (Fe)	87.49	10.05	-95.12
MG (acetate)	83.56	3.50	-3.88
MG (H_2)	76.08	-8.97	-16.35
MT (O_2)	85.89	7.38	-102.24
MT (NO_3^-)	85.89	7.38	-58.63
MT (Fe)	85.89	7.38	-97.79
MT (SO_4^{2-})	85.89	7.38	-2.67

ΔG_n represents Gibbs free energy change for N_2 fixation, calculated using the equation below (Equation S1). 100 $kJ/e^{-}eq$ represents the energy from ATPs, while $\Delta G_c^{0'}$ is the Gibbs free energy change for Rd (Table S5, listed in column $\Delta G^{0'}$). ε is energy transfer efficiency.

$$\Delta G_n = 100 - \Delta G_c^{0'} \times \varepsilon \qquad \text{Equation S1}$$

Table S5. Gibbs free energy under the standard condition for the Rd and Ra half reactions (1)

Half chemical reaction	$\Delta G^{0'}(kJ/e^{-}eq)$
$\frac{1}{8}\ H_2S + \frac{1}{2}\ H_2O = \frac{1}{8}SO_4^{2-} + \ \frac{5}{4}H^+ + e^-$	-20.85
$\frac{1}{8}\ CH_3COO^- + \frac{3}{8}\ H_2O = \frac{1}{8}CO_2 + \frac{1}{8}HCO_3^- + e^- + H^+$	-27.40
$\frac{1}{2}\ H_2 = H^+ + e^-$	-39.87
$\frac{1}{4}\ H_2O + \frac{1}{8}\ CH_4 = \frac{1}{8}\ CO_2 + \ H^+ + e^-$	-23.52
$\frac{1}{4}\ O_2 + \ H^+ + e^- = \ \frac{1}{2}\ H_2O$	-78.72
$\frac{1}{8}NO_3^- + \frac{5}{4}H^+ + e^- = \frac{1}{8}NH_4^+ + \frac{3}{8}\ H_2O$	-35.11

$Fe^{3+} + e^- = Fe^{2+}$	-74.27
$\frac{1}{8} CO_2 + H^+ + e^- = \frac{1}{4} H_2O + \frac{1}{8} CH_4$	23.52
$\frac{1}{8} SO_4^{2-} + \frac{5}{4} H^+ + e^- = \frac{1}{8} H_2S + \frac{1}{2} H_2O$	20.85

Table S6. Values for A, f_S, f_e

Model Types	A	f_S	f_e
SO (O_2)	0.92	0.52	0.47
SO (NO_3^-)	1.62	0.38	0.62
SO (Fe)	0.96	0.51	0.49
MG (acetate)	19.30	0.05	0.95
MG (H_2)	3.50	0.22	0.78
MT (O_2)	0.83	0.55	0.45
MT (NO_3^-)	1.44	0.41	0.59
MT (Fe)	0.86	0.54	0.46
MT (SO_4^{2-})	31.64	0.03	0.97

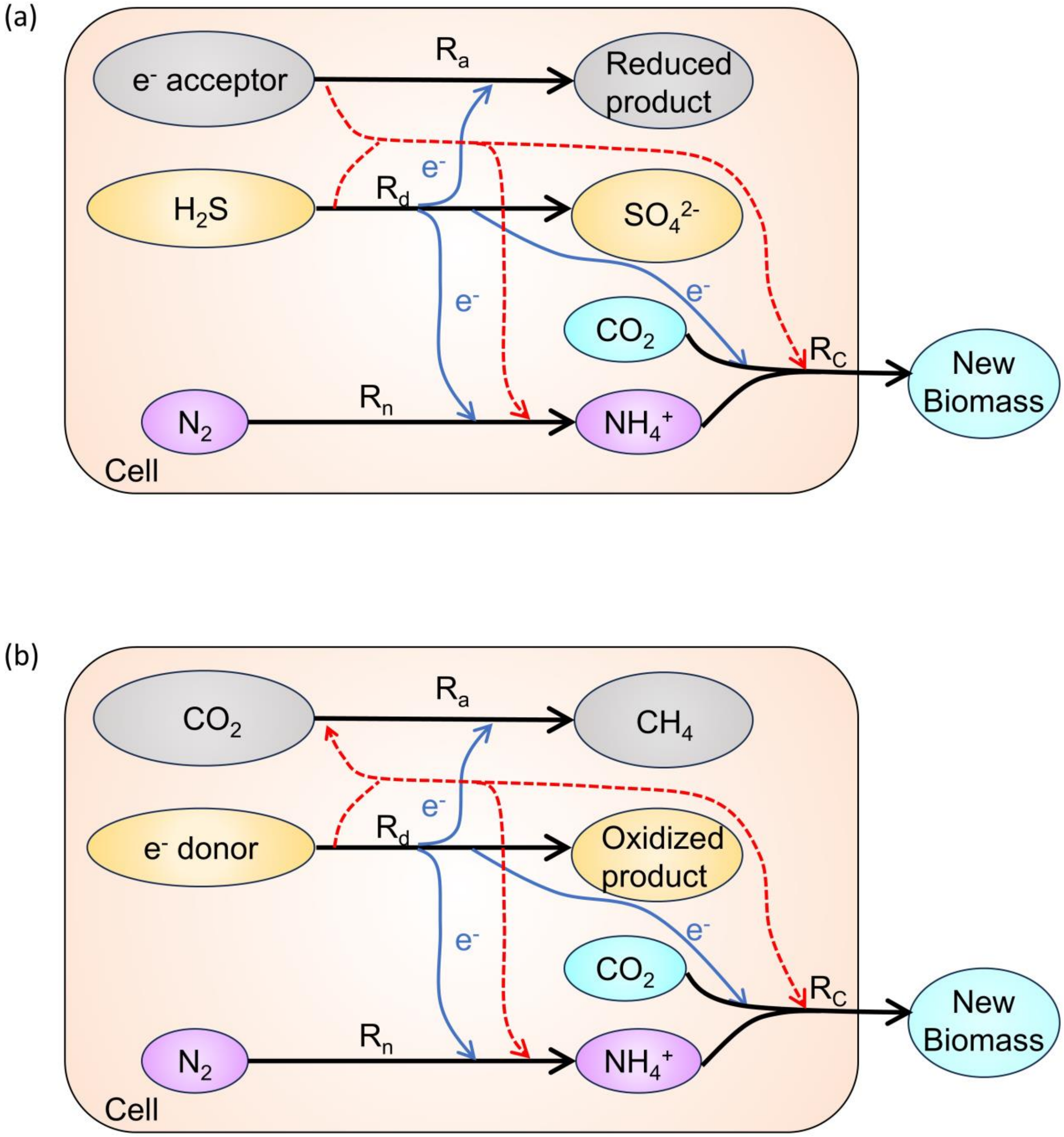
(a)
Ra
e- acceptor
Reduced product
e-
H2S
Rd
SO42-
e-
e-
CO2
RC
New Biomass
N2
Rn
NH4+
Cell
(b)
Ra
CO2
CH4
e-
e- donor
Rd
Oxidized product
e-
e-
CO2
RC
New Biomass
N2
Rn
NH4+
Cell

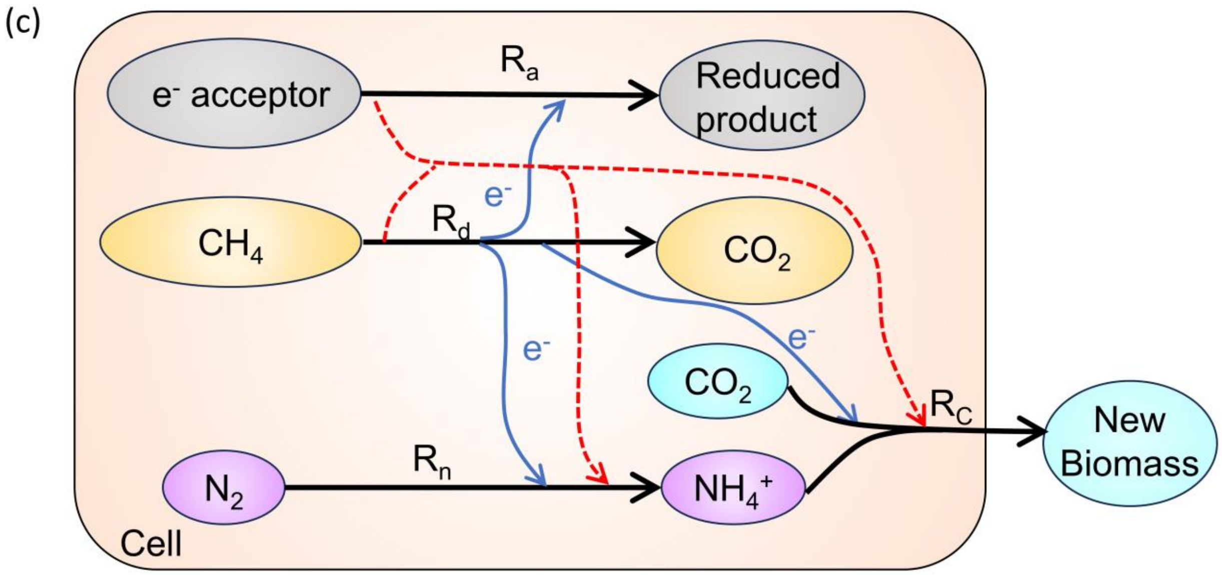

Figure S1. Summary of different CFM-CFN model types. Mechanisms of sulfide oxidizers (a), methanogens (b), and methanotrophs (c). Black arrows are reactions, blue arrows are electron flow, and red dash arrows are energy flow.

Derivation of equation [12]

Here, we explain the derivation of the equation [12] in the main text. In equation S2, ΔG_s means the energy required to synthesize one equivalent of cells from a given carbon source. To calculate ΔG_s, the first step is to determine the energy change resulting from the conversion of the carbon source to the organic intermediates for cell synthesis. Here the common organic intermediates can be activated acetate (i.e., acetyl-CoA). We used equation S3 to calculate the energy required to convert the carbon source to activated acetate (ΔG_p). In equation S3, we used the reduction free energy of activated acetate (30.9 $kJ/e^{-}eq$ (1)) minus Gibbs free energy for electron donation ($\Delta G_c^{0'}$ for Rd, listed in Table S5) to calculate ΔG_p. For different models, we have different ΔG_p based on equation S3.

After the synthesis of activated acetate, the organic carbon is converted into cellular carbon. The energy required here (Equation S2, ΔG_{pc}) is 18.8 $kJ/e^{-}eq$. Then we considered energy-transfer efficiency ε. And we used an exponent n to represent accounts for the fact of ΔG_p for some electron donors: when ΔG_p is negative, meaning energy is obtained by its conversion to activated acetate, n equals -1. When ΔG_p is positive, meaning energy is required from activated acetate synthesis, n equals 1.

$$\Delta G_s = \frac{\Delta G_p}{\varepsilon^n} + \frac{\Delta G_{pc}}{\varepsilon} \qquad \text{Equation S2}$$

$$\Delta G_p = 30.9 - \Delta G_c^{0'} \qquad \text{Equation S3}$$

After we calculated how much energy is needed to synthesize (ΔG_s). We can estimate how much energy can be provided from the electron donors. Firstly, we defined that A equivalents of electron must be lost from electron donors. For each equivalent of lost electrons, ΔG_r is the free energy released from oxidation, which can be calculated from the difference of $\Delta G^{0'}$ between Rd and Ra. So, the total energy released from electron donation is $A\Delta G_r$. Different models have different ΔG_r (listed in Table S4). Then we multiplied $A\Delta G_r$ by energy transfer efficiency ε and get the $A\varepsilon\Delta G_r$, which means the energy transferred to the carrier. During the transfer process, some amount of energy is lost because of the inefficiency.

Based on the energy balance, the energy provided and used should equals to each other, so the right term of Equation S4 should be 0:

$$A\varepsilon\Delta G_r + \Delta G_s = 0 \qquad \text{Equation S4}$$

Then we left term A on the left and moved all the other terms to the right. And get Equation S5.

$$A = -\frac{\Delta G_s}{\varepsilon\Delta G_r} \qquad \text{Equation S5}$$

In addition to the common biosynthesis process (inorganic C to activated acetate to biomass), in this model, we also considered N_2 fixation as one of the maintenance pathways. This also needs electron donors to be oxidized to supply energy and electrons. So, we separated ΔG_s into two parts. ΔG_n means energy requirements for N_2 fixation. We calculated it by using Gibbs free energy difference between electron donation half-reaction (Rd) and N_2 fixation half-reaction (Rn) (Equation S1). Different ΔG_n values are listed in Table S4. Here we multiplied ΔG_n with f_n (calculated from main text equation [16]), which means the fraction of N_2 fixation in the sum of N_2 fixation and biosynthesis. For biosynthesis, we multiplied ΔG_s by f_c, which means the fraction of biosynthesis in the sum of N_2 fixation and biosynthesis.

$$A = -\frac{(f_n \times \Delta G_n + f_c \times \Delta G_s)}{\varepsilon \Delta G_r} \qquad \text{Equation S6}$$

Finally, substituting Equation S1 into Equation S5 yields Equation S6, which matches Equation [12] in the main text.

$$A = -\frac{(f_n \times \Delta G_n + f_c \times \left(\frac{\Delta G_p}{\varepsilon^n} + \frac{\Delta G_{pc}}{\varepsilon}\right))}{\varepsilon \Delta G_r} \qquad \text{Equation S7}$$

Derivation of equation [13] and [14]

When microorganisms use an electron-donor substrate for synthesis, a portion of its electrons (f_e) is initially transferred to the electron acceptor to provide energy. Other portions of electrons can be transferred to growth and maintenance (f_S). The sum of f_e and f_s is 1.

$$f_S + f_e = 1 \qquad \text{Equation S8}$$

Based on Equations S4 and S5, the energy from biosynthesis should be *A* times the energy from electron donation-acceptance, so here, the ratio of electrons in electron donation-acceptance should be *A* times the electron goes to biosynthesis (Equation S9).

$$f_e : f_S = A \qquad \text{Equation S9}$$

Then we transform Equation S9 and have Equation S10.

$$f_e = A \times f_S \qquad \text{Equation S10}$$

Substituting equation S10 into Equation S8 yields Equation S11.

$$f_S + A \times f_S = 1 \qquad \text{Equation S11}$$

Then we keep f_S in the left side and move everything else to the right side. Then we got S12 which is equation [13] in the main text.

$$f_S = \frac{1}{1+A} \qquad \text{Equation S12}$$

Substituting Equation S12 to Equation S10 yields Equation S13, which is equation [14] in the main text.

$$f_e = A \times f_S = \frac{A}{1+A} \qquad \text{Equation S13}$$

Add the pH and temperature effect

Here, we used Equation S14 to simulate the effect of pH and temperature. For each of the chemical reactions we used in each model, we calculated their ΔG (here means Gibbs free energy changes when the pH and temperature are not in standard conditions: pH = 7, temperature (°C) = 25). $\Delta G^{0'}$ means Gibbs free energy changes under standard conditions. T is the temperature (unit: K, equals to °C + 273.15K). The $T/298.15$ means a term for temperature change. In the term ($RTn_{H^+} \times \ln\frac{10^{-7}}{[H^+]}$), R is a conversion constant, and the value is 8.314 J/mol K. n_{H^+} is the coefficient of H^+ in the chemical formulas, if H^+ is reactant, then use the positive value. If H^+ is on the product side, then use the negative value. $[H^+]$ is the concentration of H^+. Based on this equation, we can simulate how temperature and pH influence the ΔG, and then adapted ΔG in the previous equations and simulate the N_2 fixation yield.

$$\Delta G = (T/298.15) \times \Delta G^{0'} + RTn_{H^+} \times \ln\frac{10^{-7}}{[H^+]} \qquad \text{Equation S14}$$

References

1. Rittmann B, McCarty PL. 2020. Environmental Biotechnology: Principles and Applications. McGraw-Hill: New York, NY.
